# Mucosal vaccination induces protection against SARS-CoV-2 in the absence of detectable neutralizing antibodies

**DOI:** 10.1038/s41541-021-00405-5

**Published:** 2021-11-29

**Authors:** Chaojie Zhong, Hongjie Xia, Awadalkareem Adam, Binbin Wang, Renee L. Hajnik, Yuejin Liang, Grace H. Rafael, Jing Zou, Xiaofang Wang, Jiaren Sun, Lynn Soong, Alan D. T. Barrett, Scott C. Weaver, Pei-Yong Shi, Tian Wang, Haitao Hu

**Affiliations:** 1 Department of Microbiology and Immunology, University of Texas Medical Branch, Galveston, TX 77555 USA; 2 Department of Biochemistry and Molecular Biology, University of Texas Medical Branch, Galveston, TX 77555 USA; 3 Department of Pathology, University of Texas Medical Branch, Galveston, TX 77555 USA; 4 Sealy Institute for Vaccine Sciences, University of Texas Medical Branch, Galveston, TX 77555 USA; 5 Institute for Human Infections and Immunity, University of Texas Medical Branch, Galveston, TX 77555 USA; 6 World Reference Center for Emerging Viruses and Arboviruses, University of Texas Medical Branch, Galveston, TX 77555 USA

**Keywords:** Biological sciences, Infectious diseases, Vaccines

## Abstract

A candidate multigenic SARS-CoV-2 vaccine based on an MVA vector expressing both viral N and S proteins (MVA-S + N) was immunogenic, and induced T-cell responses and binding antibodies to both antigens but in the absence of detectable neutralizing antibodies. Intranasal immunization with the vaccine diminished viral loads and lung inflammation in mice after SARS-CoV-2 challenge, which correlated with the T-cell response induced by the vaccine in the lung, indicating that T-cell immunity is also likely critical for protection against SARS-CoV-2 infection in addition to neutralizing antibodies.

SARS-CoV-2 is the cause of the disease COVID-19^1^ that is currently a pandemic involving more than 170 million human infections and 3.8 million deaths worldwide. A large number of COVID-19 vaccine candidates based on various platforms are in development. Some of these vaccines have shown promising clinical efficacy, including two mRNA vaccines and a human Adenovirus 26 viral vector vaccine that were approved by the FDA for use in the U.S. under Emergency Use Authorization^2,3^.

Nearly all candidate COVID-19 vaccines utilize the viral spike protein (S) or a subunit of the protein for induction of protective immunity. Many are proposing neutralizing antibodies as a correlate of protection^2,3^. The role of vaccine-induced immune parameters other than neutralizing antibodies for protection from SARS-CoV-2 infection is less clear. Available evidence indicates that T cells may play a role in immune control of coronavirus infections^4–9^. In this study, we report a multigenic SARS-CoV2 vaccine based on the modified vaccinia ankara (MVA) vector that expresses both viral nucleocapsid (N) and spike (S) (MVA-S + N). We demonstrate that the vaccine is immunogenic but does not induce detectable neutralizing antibodies. Intranasal immunization with the vaccine-induced significant protection in a mouse model after SARS-CoV-2 challenge, which correlated with T-cell response in the lung induced by the vaccine.

In order to generate recombinant MVA-S+N vaccine, viral S (USA-WA1/2020; wide-type; no pre-fusion stabilizing mutations) and N genes were cloned into two transfer plasmids, pLW17 and pLW9, respectively^10^, to construct pLW17-S and pLW9-N (Fig. 1a). To aid recombinant MVA purification, the S and N genes were linked to mNeonGreen and mScarlet reporter, respectively, through a self-cleavage site, P2A^11^. The strategy for generating MVA-S + N virus is shown in Supplementary Fig. 1a. BHK-cells were infected with wild-type MVA for 2 h, followed by co-transfecting the cells with the constructed pLW17-S-mNeonGreen and pLW9-N-mScarlet plasmids. MVA-S + N virus was generated through homologous recombination. Cells co-expressing mNeonGreen and mScarlet reporters after transfection were confirmed by fluorescence microscope (Supplementary Fig. 1b). To purify the recombinant MVA, two rounds of cell sorting were performed to isolate the double-positive cells (Supplementary Fig. 1c), followed by plaque purifications^12^. Purified MVA-S + N was then propagated and titrated^12^. Expression of SARS-CoV-2 S and N proteins in cells by MVA-S + N was confirmed by western blotting (Fig. 1b).

**Fig. 1:**
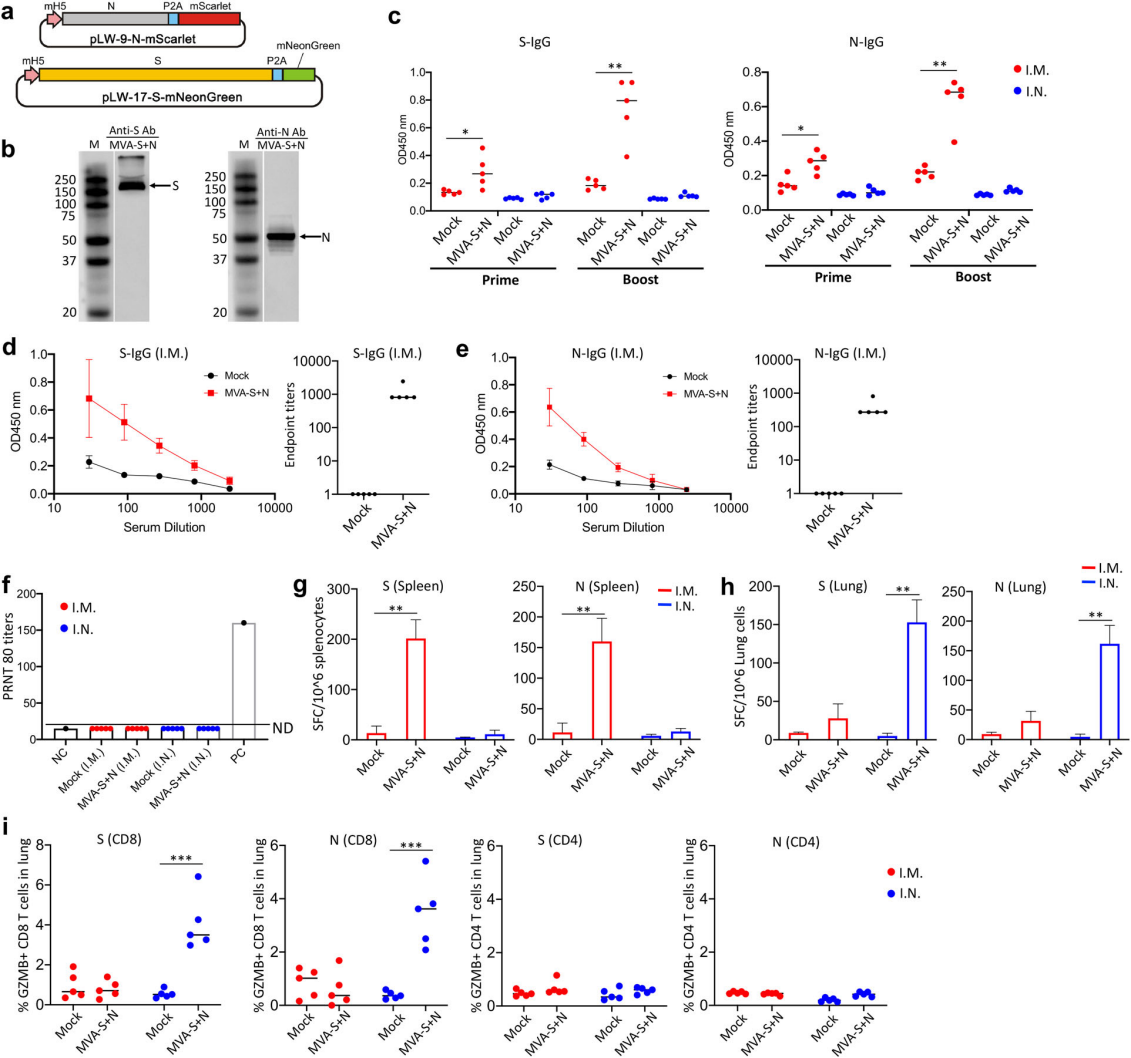
Vaccine generation and immune assessment. **a** Vaccine cloning. SARS-CoV-2 N or S gene was respectively cloned into MVA transfer plasmid pLW9 and pLW17. The N and S insert sequences were respectively linked to mScarlet or mNeonGreen reporter for recombinant virus purification. A P2A sequence was introduced between the viral gene (S or N) and reporter sequence for cleavage after protein expression. **b** WB confirmation of SARS-CoV2 S and N protein expression in cells infected with the vaccine. BHK-21 cells were infected with purified MVA-S + N for 48 h. Proteins were extracted from the infected cells for WB analysis using specific antibody for S (GTX632604) or N (MA5-29981). Blots shown were derived from the same experiment and were processed in parallel. **c** Vaccine-induced serum binding IgG after I.M. (red) and I.N. (blue) immunization. S- and N-specific binding IgG in sera of mock- and vaccine-immunized mice collected after prime or boost vaccination were measured by ELISA. The data were shown as OD450 values (serum dilution: 1:30). ELISA was conducted in duplicate and mean OD value for each sample was used. **d**, **e** S-specific **d** or N-specific **e** binding IgG in serially diluted sera (left) and IgG endpoint titers (right) from mice at 2 weeks after boost vaccination. Serum samples were 1:3 serially diluted (initial dilution: 1:30) and binding IgG in the diluted samples was quantified by ELISA. Data (left panel of **d** or **e**) were shown as mean OD450 nm values for each group (*n* = 5). IgG endpoint titers for S (**d**; right panel) or N (**e**; right panel) were also shown. **f** Serum SARS-CoV2 neutralizing activity after I.M. (red) or I.N. (blue) immunization (at 2 weeks after boost immunization). Neutralizing activity was measured by plaque reduction neutralization test (PRNT). Neutralizing titers (PRNT80) are compared between the mock and vaccinated groups. Negative and positive controls are included. **g** IFN-γ ELISPOT measurement of S-specific (left) or N-specific (right) T cells in the mouse spleen after I.M. (red) or I.N. (blue) immunization. Cells harvested at 2 weeks after boost vaccination were measured. **h** IFN-γ ELISPOT measurement of S-specific (left) or N-specific (right) T cells in the mouse lung after I.M. (red) or I.N. (blue) immunization. **i** Intracellular cytokine staining (ICS) and flow cytometric analysis of S- and N-specific CD8 and CD4 T cells in the mouse lung. Frequencies of GZMB-expressing CD8 and CD4 T cells in the lung between the control and vaccinated mice after I.M. (red) and I.N. (blue) immunization. In this figure, error bars (d,e,g,h) showed standard deviation (SD) within each group. *p < 0.05; **p < 0.01, ***p < 0.001; unpaired Student’s *t* test.

Next, vaccine-induced immune responses were evaluated in mice following intramuscular (I.M.) or intranasal (I.N.) immunization. I.N. immunization was also tested since mucosal immunity is considered important for protection against SARS-CoV-2 infection. Two groups of WT Balb/c mice were prime-boost vaccinated with PBS (mock) or MVA-S + N (10^7^ pfu) at week 0 and week 3 via the intramuscular route (I.M.). Another two groups of mice received the same mock or MVA vaccine, respectively, via the intranasal route (I.N.) (Supplementary Fig. 2a). Sera were collected after prime vaccination to analyze the antibody response; two weeks after boost vaccination (week 5), all mice were euthanized; blood and tissue samples were collected to analyze antibody and cellular responses. Compared to mock-immunized mice, I.M. immunization with MVA-S + N rapidly induced detectable, albeit at low levels, of binding IgG at seven days after prime vaccination; S- and N-specific binding IgG was detected at comparable levels (Fig. 1c). Compared to prime vaccination, boost vaccination enhanced the levels of both S- and N-specific binding IgG in the sera (Fig. 1c). Different from I.M. immunization, no or very little serum S- or N-specific binding IgG was detected in mice after I.N. immunization (OD values < 0.15 and comparable with mock group) (Fig. 1c). Endpoint titers (EPTs) for serum binding IgG after boost vaccination in the I.M. groups were measured (Fig. 1d, e). Sera were serially diluted and levels of S-specific (Fig. 1d) or N-specific (Fig. 1e) binding IgG in the serially diluted samples was examined by ELISA to determine EPTs. The data showed that compared to the mock group, EPTs for serum S- and N-specific IgG were observed in the vaccine group with median value of 810 and 270, respectively (Fig. 1d, e).

Serum neutralizing activity was also measured by Plaque Reduction Neutralizing Tests (PRNT)^13^ using live SARS-CoV2 virus. For I.M. immunization, while the vaccine-induced significant levels of serum binding IgG to S protein, no significant neutralizing activity was detected in any of the vaccinated mice in sera at 2 weeks post the booster vaccination (PRNT80 < 20 and comparable with NC) (Fig. 1f). Similarly, no neutralizing activity was detected either in the sera of I.N. immunized mice (Fig. 1f), which was as expected and consistent with no detection of binding IgG in sera of the I.N. groups (Fig. 1c).

Next, we examined vaccine-induced cellular immune response in mice following I.M. and I.N. immunization. First, vaccine-specific, systemic T-cell response in the spleen was measured using IFN-γ T-cell ELISPOT (Fig. 1g). For the I.M. groups, we observed that MVA vaccination induced significant levels of S- and N-specific T cells in the spleen (mean SFC/10^6^ cells for S: 201.5 in vaccine vs. 13.5 in mock; mean SFC/10^6^ cells for N: 160 in vaccine vs. 11.5 in mock) (*p* < 0.01 for S and N) (Fig. 1g). However, distinct from I.M. immunization, I.N. immunization induced no or little vaccine-specific T-cell response in the spleen (mean SFC for S: 11.2 in vaccine vs. 4.8 in mock; mean SFC for N: 13 in vaccine vs. 5.6 in mock (Fig. 1g). This was not surprising and consistent with inefficiency of I.N. immunization to induce antibody response in sera (Fig. 1c). Together, these data support that the MVA-S+N vaccine is immunogenic and induces systemic antibody response (binding IgG) and T-cell response for both S and N proteins in mice following I.M. immunization, although no detectable neutralizing activity was induced. In contrast, I.N. immunization with the vaccine did not appear to induce significant levels of systemic antibody and T-cell responses.

Since mucosal immunity in the respiratory system is considered critical for protection against SARS-CoV-2 infection, we also measured vaccine-specific antibody and T-cell responses in the lung and compared them between I.M. and I.N. immunizations. To determine the levels of antibody production in the lung, bronchoalveolar lavage (BAL) samples were harvested from all mice and vaccine-specific binding antibodies were examined by ELISA. We observed that that I.M. immunization induced significant levels of N- and S-specific binding IgG in the BAL (mean OD value for S: 0.05 in mock vs. 0.67 in vaccine; mean OD value for N: 0.02 in mock vs. 0.65 in vaccine) (*p* < 0.001 for S and N) (Supplementary Fig. 2b). Similar to I.M immunization, I.N. immunization also induced significant levels of S- and N-specific binding IgG in the BAL (mean OD value for S: 0.02 in mock vs. 0.46 in vaccine; mean OD value for N: 0.02 in mock vs. 0.45 in vaccine) (*p* < 0.01 for S and N) (Supplementary Fig. 2b). However, no or very little binding IgA for S and N was detected in the BAL for both I.M. and I.N. immunization (OD values < 0.15 and comparable with the mock group) (Supplementary Fig. 2b).

We next quantified vaccine-specific T-cell response in the lung by IFN-γ T-cell ELISPOT. Representative ELISPOT data were shown in Supplementary Fig. 2c. Intriguingly, while I.M. immunization induced S- and N-specific T-cell responses in the spleen as descried above (Fig. 1g), it did not elicit significant T-cell response in the lung (mean SFC for S: 28 in vaccine vs. 9 in mock; mean SFC for N: 31 in vaccine vs. 10 in mock) (*p* > 0.05 for S and N) (Fig. 1h). In contrast, I.N. immunization induced high levels of S- and N-specific T-cell responses in the lung following I.N. immunization (mean SFC for S: 152 in vaccine vs. 5 in mock; mean SFC for N: 161 in vaccine vs. 5 in mock) (*p* < 0.001 for S and N) (Fig. 1h), although it did not effectively induce the T-cell responses in the spleen as described above (Fig. 1g).

To further characterize the vaccine-induced T cells in the lung, intracellular cytokine staining (ICS) and flow cytometric analysis were performed. Cells isolated from mouse lungs were ex vivo-stimulated with overlapping peptide pools spanning SARS-CoV2 N or S protein, followed by cell surface staining for lineage markers and intracellular staining for granzyme B (GZMB). Gating strategy and representative flow cytometric plots were shown in Supplementary Fig. 2d. As shown, while there were low, basal levels of GZMB expression in un-stimulated CD8 + T cells (NC) (~1.47%), cell stimulation with the recall peptides (N or S peptides) induced significant GZMB up-regulation in CD8 T cells (N peptides: 5.11%; S peptides: 3.98%) (Supplementary Fig. 2d). Cumulative analysis demonstrated that for I.N. immunization, compared to the mock group, MVA vaccination induced robust GZMB-expressing CD8 + T cells specific for both N and S proteins in the lung (*p* < 0.001 for S and N) (Fig. 1i), indicating cytotoxic potential of these cells. In comparison to CD8 + T cells, vaccine-specific GZMB + CD4 T cells in the lung were low and did not differ significantly between the mock and vaccine groups (Fig. 1I). In contrast to I.N. immunization, I.M. immunization with the vaccine induced no or low levels of vaccine-specific GZMB + CD8 T-cell response in the lung (Fig. 1i). Taken together, these data indicate that differential induction of vaccine-specific T-cell immunity in the lung, especially the GZMB-expressing CD8 T cells, is a key difference between I.N. and I.M. immunization.

Lastly, we comparatively evaluated vaccine-induced protection after I.N. and I.M. immunization in SARS-CoV-2-challenged mice. Immunization timelines were identical to those described for the immunogenicity studies. At week 5, all mice were intranasally challenged with a mouse-adapted SARS-CoV-2 strain^14^, followed by euthanasia at two days after challenge for analyses of viral loads and inflammation in the lung (Supplementary Fig. 3). Expression of three SARS-CoV2 viral RNAs (S, E, and RdRp) in the lung were measured by qRT-PCR as an indication of viral loads. The data were analyzed using the standard delta-delta Ct (2^−^^∆∆Ct^) method^15^ and were shown as fold change relative to the mock control of I.N. immunization (Fig. 2a). For I.N. immunization, we observed that MVA vaccination significantly reduced viral loads in the lung of all the vaccinated mice compared to the mock group (S: > 11-fold; E: > 12-fold; RdRp: >9-fold) (*p* < 0.0001 for S and E, *p* < 0.001 for RdRp) (Fig. 2a). In contrast, no significant protection was observed by I.M. immunization (Fig. 2a). Next, we examined lung inflammation after immunization and SARS-CoV-2 challenge. Lung RNA samples from the above I.N. or I.M. groups were examined for inflammatory gene expression by qRT-PCR (Fig. 2b). For each immunization route, the corresponding mock and vaccine groups without SARS-CoV-2 challenge were included as baseline for normalization (Fig. 2b). First, regardless of immunization route, we observed that SARS-CoV-2 challenge induced substantial up-regulation of inflammatory gene expression compared to the no-challenge groups. Among the genes examined, CCL7, CXCL10, and CCL2 were abundantly up-regulated, while CCL3, TNF-α, and IL-6 were modestly up-regulated (Fig. 2b). For I.N. immunization, compared to the mock group, MVA vaccination markedly diminished the expression of 5 out of the 6 inflammatory genes (CCL2, CCL3, CCL7, CXCL10, and TNF-α) in the lung, except IL-6 (Fig. 2b). In contrast, for I.M. immunization, expression of these genes was comparable between the mock and vaccine groups, indicating that I.M. immunization with the vaccine did not significantly diminish SARS-CoV-2-induced inflammation in the lung (Fig. 2b). Together, the data indicate that intranasal delivery of the MVA-S + N vaccine reduces viral loads and virus-induced inflammation in the mouse lung.

**Fig. 2:**
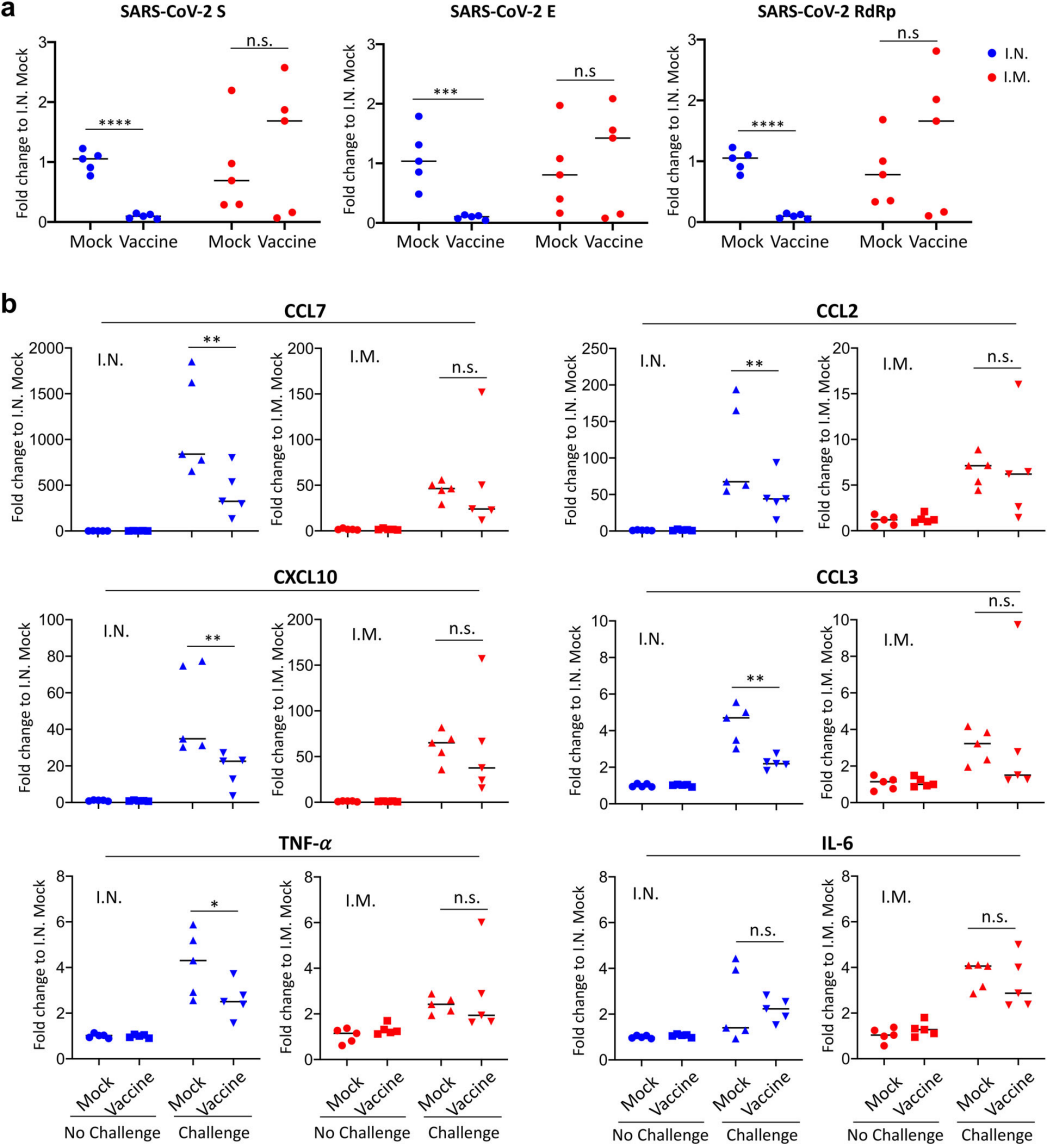
Analysis of viral loads and lung inflammation following SARS-CoV-2 challenge. **a** Comparison of levels of SARS-CoV2 viral RNAs in the lung between I.N. (blue) and I.M. (red) immunization groups following SARS-CoV-2 challenge. Expression of individual viral RNAs (S, E, RdRp) was first normalized to mouse GAPDH and then shown as fold change relative to the mock group of I.N. immunization. **b** Measurement of lung inflammatory gene expression in I.N. (blue) or I.M. (red) immunized mice. For each immunization route, lung tissue RNA was extracted from four groups of mice (mock/no challenge, vaccine/no challenge, mock/challenge, and vaccine challenge) and then subjected to qRT-PCR quantification of mouse inflammation genes (CCL7, CCL2, CXCL10, CCL3, TNF-α, and IL-6). Gene expression was first normalized to mouse GAPDH and then compared among the four groups within each immunization route. The data were shown as fold change in RNA copies relative to the corresponding mock/no challenge group. *p < 0.05; **p < 0.01; ***p < 0.001; ****p < 0.0001; n.s. non-significant. unpaired Student’s *t* test.

In summary, we described a multigenic SARS-CoV-2 vaccine (MVA-S + N) that was immunogenic and induced specific T-cell and binding antibody responses in mice. In our study, it remains unclear why the vaccine is ineffective in inducing neutralizing antibodies. We speculate that this is likely related to either expression of S gene with the reporter as a fusion protein, which may affect its neutralizing epitopes in vivo, or lack of pre-fusion stabilizing mutations^16–18^ in the S gene in our vaccine construct. The mechanism needs to further explored in future studies. However, this finding provided an opportunity to explore parameters of immune protection in addition to neutralizing antibodies. Indeed, our study presented evidence that intranasal immunization with the MVA-S + N vaccine induced some protection against SARS-CoV-2, which correlated with the T-cell response in the lung.

Our study has implications for SARS-CoV-2 vaccine development. Given the constant mutations of S protein, including the generation of SARS-CoV-2 spike variants with partial escape from vaccine-induced neutralizing antibodies^19,20^, it is reasonable to propose that simultaneous targeting of S protein and another conserved antigen of the virus may induce neutralization-independent protection and confer some cross protection against variants. In future studies, it would be interesting to compare our approach with those targeting S alone for vaccine-induced protection against SARS-CoV-2 variants. In addition, with increased identification of immunodominant epitopes of SARS-CoV-2, vaccine constructs that express the antigenic fragments of S and N proteins should also be explored in the future. To summarize, our present study indicates that T-cell immunity is also critical for protection against SARS-CoV-2 in addition to neutralizing antibodies, and is likely an important consideration for SARS-CoV-2 and pan-coronavirus vaccine development.

## Methods

### Recombinant vaccine construction

The spike (S) and nucleocapsid (N) genes of SARS-CoV2 were amplified from the infectious cDNA clone of 2019-nCoV/USA-WA1/2020 strain^21^, fused with a gene cassette of porcine teschovirus-1 2 A (P2A) and a fluorescent marker (S gene with mNeonGreen and N gene with mScarlet). Gene insertions were respectively cloned to transfer plasmid pLW17 or pLW9 (kindly provided by Dr. Bernard Moss) by using NEBuilder HiFi DNA Assembly mix (Cat #: E2621; NEB) to generate plasmid constructs pLW17-S-mNeonGreen and pLW9-N-mScarlet.

### Vaccine generation and purification

Recombinant MVA encoding SARS-CoV2 S and N genes were generated using a protocol involving flow cytometry-based cell sorting and plaque purification^22^. Briefly, monolayers of BHK-21 were grown in complete DMEM medium in six-well culture plates to 80% confluency. Cells were then infected with wild-type MVA (VR-1508; ATCC) at 0.01 multiplicity of infection (MOI) for 2 h, followed by co-transfection with plasmids pLW17-S-mNeonGreen and pLW9-N-mScarlet using Lipofectamine 3000 Transfection Kit (Cat#: L3000-015; Invitrogen). 48 h after transfection, cells co-expressing mNeonGreen and mScarlet in the culture plate were confirmed by fluorescence microscope (Fig. 1c). Cells were then harvested and sort purified for mNeonGreen and mScarlet double-positive population by the BD FACS Sorter (UTMB flow cytometry and cell-sorting core). Lysates of sorted cells were used to further purify recombinant MVA encoding both S and N (MVA-S + N) by using the plaque purification protocol^22^ (4-5 rounds based on mNeonGreen and mScarlet marker). Purified MVA-S + N virus was propagated in BHK-21 cells, followed by viral concentration and titration^22^.

### Vaccine in vitro characterization

Purified MVA-S + N vaccine was first characterized in infected BHK-21 cells by using fluorescence microscope. Monolayers of BHK-21 cells at 80% confluency were infected with plaque purified MVA-S + N (MOI = 1) for 48 h. Co-expression of mNeonGreen and mScarlet in the infected cells was examined by fluorescence microscope. In addition, the vaccine was characterized for SARS-CoV2 S and N protein expression in the infected BHK-21 cells by western blot. Briefly, BHK-21 cells were infected with empty MVA (MOI = 1), MVA-S + N (MOI = 1), or not infected, for 48 h. Infected cells were lysed in RIPA buffer (Thermo Fisher Scientific) and kept on ice for 15 min. Cell lysates were centrifuged and the supernatants were collected for quantification of total protein concentration using Microplate BCA Protein Assay Kit (Pierce™, Thermo Fisher Scientific). Equivalent amounts of protein were separated by SDS-PAGE using precast 4–15% SDS polyacrylamide gels (Bio-Rad). Proteins were subsequently transferred onto a nitrocellulose membrane (Bio-Rad). The membrane was blocked in tris buffered saline (TBS) containing 0.05% Tween-20 (Thermo Fisher Scientific) and 5% (w/v) non-fat dried milk (Bio-Rad) for 1.5 h at room temperature, followed by incubation with anti-SARS-CoV2 spike mouse mAb (GTX632604, GeneTex; 1:500) or anti-SARS-CoV2 nucleocapsid mouse mAb (MA5-29981, Invitrogen; 1:1000) for overnight at 4 °C. After washing in TBST (3 times for 5 min), the membrane was incubated for 1 h with HRP-linked anti-mouse IgG (7076 S, Cell Signaling; 1:5000). The membrane was washed, and proteins were visualized using the ECL Western Blotting Substrate (Thermo Fisher Scientific). Uncropped WB images were shown in Supplementary Fig. 1d. All blots were derived from the same experiment and were processed in parallel.

### Animal ethics statement

Animal study was conducted in accordance with the recommendations in the Guide for the Care and Use of Laboratory Animals of the National Institutes of Health. Animal protocol was approved by the Institutional Animal Care and Use Committee (IACUC) at the University of Texas Medical Branch (UTMB).

### Mouse immunization, sample collection, and immunogenicity

Animal study design and experimental timelines were summarized in various figures of the manuscript. Briefly, 6-week-old female BALB/c mice were obtained from the Jackson Laboratories (Wilmington, MA, USA) and were housed in the animal facility at the Medical Research Building of the University of Texas Medical Branch. Mice (5 per group) were immunized intramuscularly (i.m.) or intranasally (i.n.) with either PBS (50 µl) as the mock control or 10^7^ PFU MVA-S + N vaccine (50 µl) using a prime-boost approach at week 0 (prime) and week 3 (boost), respectively. For immunogenicity studies, blood/serum samples were collected from all mice 1 week after prime (1st) vaccination to measure antibody response. Two weeks after the 2nd vaccination (week 5), mice were euthanized. Blood/serum, spleen, and lung tissues were collected for immune analyses. Bronchoalveolar lavage (BAL) was also collected by washing the lung with 1 ml ice-cold Dulbecco’s phosphate-buffered saline (DPBS) by using a blunt-ended needle as previously reported^23^. BAL was used for quantifying vaccine-induced antibody response in lung.

### Binding IgG and IgA by ELISA

ELISA was used to measure N- and S-specific binding IgG and IgA in sera and in BAL. ELISA plates (Greiner bio-one) were coated with 1 µg/ml recombinant S (S1 + S2-ECD; 40589-V08B1; Sino Biological) or N protein (40588-V08B; Sino Biological) in DPBS overnight at 4 °C. Plates were washed three times with wash buffer (DPBS with 0.05% Tween 20), 5 min for each time, and then blocked with 8% FBS in DPBS for 1.5 h at 37 °C. Plates were washed and incubated with serially diluted sera in blocking buffer at 50 µl per well for 1 h at 37 °C. For quantification of binding antibodies in BAL, collected BAL fluids were used for incubation without dilution. ELISA was conducted in duplicate. Plates were again washed and incubated with horse radish peroxidase (HRP) conjugated anti-mouse IgG secondary antibody (Biolegend) (1:5000) for 1 h at 37 °C. After final wash, plates were developed using TMB 1-Component Peroxidase Substrate (Thermo Fisher), followed by termination of reaction using the TMB stop solution (Thermo Fisher). Plates were read at 450 nm wavelength within 30 min by using a Microplate Reader (BioTek).

### Neutralization assay

Neutralizing activity was examined by a standard Plaque Reduction Neutralization Test (PRNT)^20,24^. The assays were performed with Vero cells using live SARS-CoV-2 at BSL-3. In brief, sera were heat-inactivated and two-fold serially diluted (dilution range of 1:10 to 1:640), followed by inculcation with 100 PFU SARS-CoV2 (USA-WA1/2020)^25^ for 1 h at 37 °C. The serum-virus mixtures were placed onto Vero E6 cell monolayer in 6-well plates for incubation for 1 h at 37 °C, followed by addition of 2-ml overlay consisting of MEM with 1.6% agarose, 2% FBS, and 1% penicillin–streptomycin to the cell monolayer. Cells were then incubated for 48 h at 37 °C, followed by staining with 0.03% liquid neutral red for 3-4 h. Plaque numbers were counted and PRNT80 were calculated. Each serum was tested in duplicates.

### IFN-γ ELISPOT

Millipore ELISPOT plates (Millipore Ltd, Darmstadt, Germany) were coated with anti-IFN-γ capture Ab (CTL, Cleveland, OH, USA) at 4 °C overnight. Splenocytes or lung mononuclear cells (0.25 × 10^6^) were stimulated in duplicates with SARS-CoV-2 S or N peptide pools (2 μg/ml, Miltenyi Biotec, USA) for 24 h at 37 °C. Cells stimulated with anti-CD3 (1 μg/ml, e-Biosciences) or medium alone were used as controls. This was followed by incubation with biotin-conjugated anti-IFN-γ (CTL, Cleveland, OH, USA) for 2 h at room temperature, and then alkaline phosphatase-conjugated streptavidin for 30 min. The plates were washed and scanned using an ImmunoSpot 4.0 analyzer and the spots were counted with ImmunoSpot software (Cellular Technology Ltd, Cleveland, OH) to determine the spot-forming cells (SFC) per 10^6^ splenocytes.

### Intracellular cytokine staining and flow cytometry

ICS was performed on single-cell suspensions isolated from lung. Briefly, equivalent portions of lung tissues were harvested from mice, minced, and digested with 0.05% collagenase type IV (Thermo Fisher Scientific) in RPMI 1640 Medium for 30 min at 37 °C. After digestion, lung single-cell suspensions were prepared by passing the lung homogenates through 70μm cell strainers. Red blood cells were removed by using Red Cell Lysis Buffer (Sigma-Aldrich). Cells (2 × 10^6^) were stimulated for 5 h at 37 °C with 1 μg/ml SARS-CoV-2 S or N peptide pool (Miltenyi Biotec) in the presence of protein transport inhibitors Golgi-stop and Golgi-plug (BD Bioscience). Cells stimulated with medium containing DMSO only or with PMA (50 ng/ml)/ionomycin (750 ng/ml) were used as negative and positive control, respectively. After stimulation, cells were stained for live/dead viability dye and surface antigens: anti-CD45-APC-Cy7 (Biolegend), anti-CD3-PE-Cy7 (Biolegend), anti-CD4-FITC (Biolegend), and anti-CD8-PerCP (Biolegend), followed by fixation and permeabilization by using BD Cytofix/Cytoperm kit (BD Bioscience). Cells were then intracellularly stained with anti-mouse GZMB-Pacific Blue (Biolegend). Samples were processed with FACS LSR-Fortessa (BD). Dead cells were excluded based on forward and side scatters and live/dead viability staining. Data were analyzed using FlowJo (TreeStar).

### SARS-CoV2 challenge and analyses of viral loads and inflammation

Two weeks after booster vaccination (either i.m. or i.n.) as described above, all mice were intranasally challenged with a mouse-adapted SARS-CoV2 CMA4 strain (2 × 10^4^ pfu)^14^. Viral challenge was conducted at the ABSL-3 facility at UTMB. Two days after challenge, all mice were euthanized and equivalent portions of lung tissues were collected for RNA extraction and viral load analysis. Total RNA was extracted from lung tissues of the SARS-CoV-2-challenged mice as well as the control unchallenged mice using the TRIzol reagent according to the manufacturer’s instructions. RNA concentration and purity were determined using the multi-mode reader (BioTek). To quantify SARS-CoV2 viral RNA and mouse inflammatory expression, cDNA was synthesized from RNA using the iScript Reverse Transcription Supermix for RT-qPCR (Bio-Rad). Expression of SARS-CoV2 (S, E, RdRp RNA) and mouse inflammatory genes (CCL2, CCL3, CCL7, CXCL10, TNF-α, and IL-1β) was quantified by qPCR using iTaq Universal SYBR Green Supermix (Bio-Rad) and the CFX Connect Real-Time PCR Detection System (Bio-Rad). Primers for individual genes were shown in the Table 1. PCR reactions (20 μl) contained 10 μM primers, 90 ng of cDNA, 10 μl iTaq universal SYBR Green supermix (2X) (Bio-Rad), and molecular grade water. PCR cycling conditions were: 95 °C for 3 min, 45 cycles of 95 °C for 5 s, and 60 °C for 30 s. For each PCR reaction, mouse GAPDH was also measured for normalization. Relative expression of target genes among different groups was calculated using the delta-delta Ct (2^−∆∆Ct^) method^15^.

**Table 1:** Primer sequences for quantitative PCR.

Target	Primer sequence
SARS-CoV2 S	F: CAGGACAAGAACACACAGGAA R: CAGGCAGGATTTGGGAGAAA
SARS-CoV2 E	F: GGAAGAGACAGGTACGTTAATA R: AGCAGTACGCACACAATCGAA
SARS-CoV2 RdRP	F: GTCATGTGTGGCGGTTCACT R: CAACACTATTAGCATAAGCAGTTGT
Mouse GAPDH	F: TTAAAAACCTGGATCGGAACCAA R: GCATTAGCTTCAGATTTACGGGT
Mouse CCL2	F: TTAAAAACCTGGATCGGAACCAA R: GCATTAGCTTCAGATTTACGGGT
Mouse CCL3	F: GTGTAGAGCAGGGGCTTGAG R: AGAGTCCTCGATGTGGCTA
Mouse CCL7	F: CCACATGCTGCTATGTCAAGA R: ACACCGACTACTGGTGATCCT
Mouse CXCL10	F: CCAAGTGCTGCCGTCATTTTC R: GGCTCGCAGGGATGATTTCAA
Mouse TNF-α	F: CTTGTTGCCTCCTCTTTTGC R: TGGTCACCAAATCAGCGTTA
Mouse IL-6	F: CTGCAAGAGACTTCCATCCAG R: AGTGGTATAGACAGGTCTGTTGG

### Statistical analysis

All statistical analyses were performed using Graph-Pad Prism 8.0. Statistical comparison between the mock and vaccine groups was performed using unpaired Student’s *t* test. The values were presented either as mean or mean ± SD where appropriate. Two-tailed *p* values were denoted, and *p* values < 0.05 were considered significant.

### Reporting summary

Further information on research design is available in the Nature Research Reporting Summary linked to this article.

## Supplementary information

Supplementary Information

Reporting Summary

## Data Availability

All data generated and/or analyzed during this study are included in this published article and its supplementary information file.
