# Supplementary material for: Mucosal vaccination induces protection against SARS-CoV-2 in the absence of detectable neutralizing antibodies
Source: NPJ Vaccines. 2021 Nov 29;6:139. doi: 10.1038/s41541-021-00405-5 (PMC8630013; doi:10.1038/s41541-021-00405-5)
Supplement: Supplementary file 1 — Supplementary Information [file 41541_2021_405_MOESM1_ESM.pdf]

Supplementary File

Mucosal vaccination induces protection against SARS-CoV-2 in the absence of detectable neutralizing antibodies

Chaojie Zhong, Hongjie Xia, Awadalkareem Adam, Binbin Wang, Renee L. Hajnik, Yuejin Liang, Grace H. Rafael, Jing Zou, Xiaofang Wang, Jiaren Sun, Lynn Soong, Alan D.T. Barrett, Scott C. Weaver, Pei-Yong Shi, Tian Wang, Haitao Hu

Supplementary Figure 1

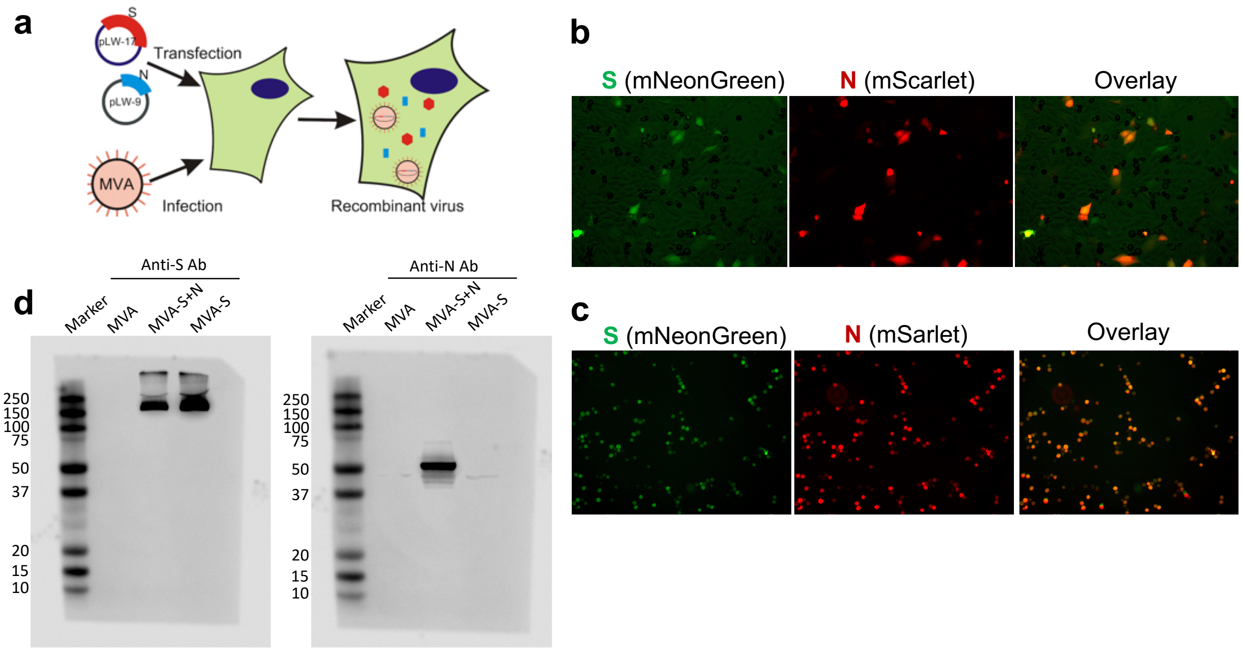

Supplementary Fig. 1. **Vaccine design, generation and characterization**. **(a)** Schematic illustration of generation of recombinant MVA expressing SARS-CoV2 S and N (MVA-S+N). BHK-cells were infected with wild-type MVA (MOI: 0.01) for 2 hours, followed by co-transfecting

cells with pLW17-S-mNeonGreen and pLW9-N-mScarlet plasmids. MVA-S+N were generated through homologous recombination and harvested 48 hours after transfection. **(b)** Confirmation for co-expression of the two reporters in the transfected cells by fluorescence microscope. Left: mNeonGreen (S), middle: mScarlet (N), right: overlay. **(c)** Microscopic confirmation of cells co-expressing mNeonGreen (S) and mScarlet (N) after FACS sorting. Transfected cells were subject to FACS sorting for mNeonGreen and mScarlet double positive population. Sorted cells were analyzed by fluorescence microscope. Left: mNeonGreen (S), middle: mScarlet (N), right: overlay. **(d)** Uncropped WB blots for Figure 1b in the main text. BHK-21 cells were infected with empty MVA, MVA-S+N, or MVA-S, as indicated, for 48 hours. Proteins were extracted from the infected cells and equal amounts of proteins were loaded in gel for WB analysis using specific antibody for S (GTX632604) or N (MA5-29981).

Supplementary Figure S2

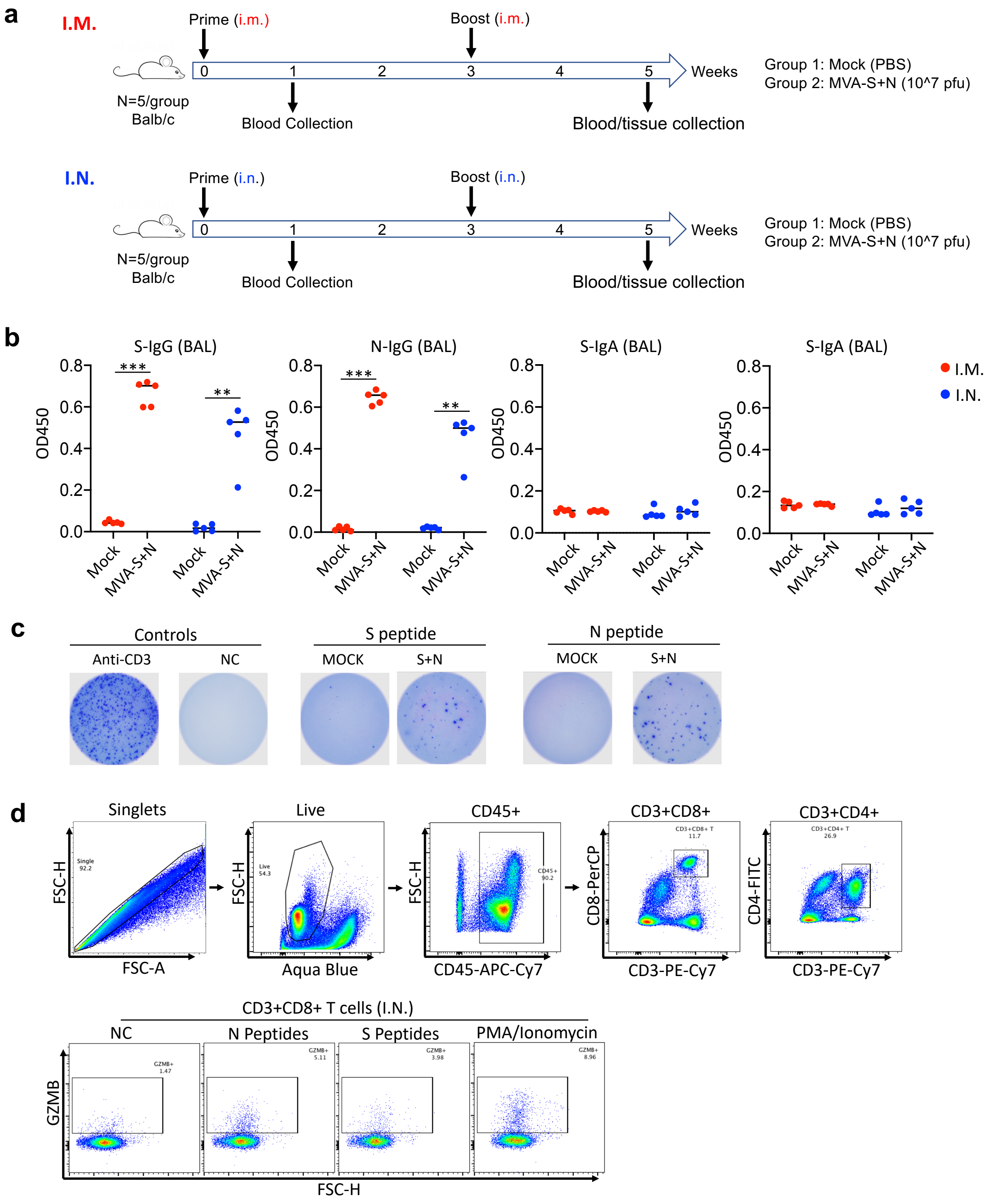

Supplementary Fig. 2. Vaccine-induced immune responses in mice following I.M. and I.N. immunization. (a) Vaccination timeline. 2 groups of BALB/c mice (n=5) were prime-boost immunized with mock (PBS) or MVA-S+N (10^7 pfu) at week 0 and 3 via I.N. route. Another 2 groups of BALB/c mice (n=5) received the same mock or MVA-S+N vaccine (10^7 pfu), respectively, via I.N. route. One week after prime vaccination (week 1), serum samples were collected for antibody analysis. Two weeks after boost vaccination (week 5), all mice were euthanized and vaccine-induced antibody and cellular immune responses were analyzed. **(b)** Comparison of S- and N-specific binding IgG and IgA in BAL from I.M. and I.N. immunized mice (no dilution). S- and N-specific antibodies were measured by ELISA. Average OD value for each sample was shown. **(c)** IFN-γ T-cell ELISPOT quantification of vaccine-specific T cells in the spleen and lung. Representative ELISPOT data for cells stimulated with recall peptides (S or N) or with mock (negative control) or anti-mouse CD3 (positive control) were shown. **(d)** ICS and flow cytometric analysis of vaccine-specific T cells in the lung. Cells of lung tissues were re-stimulated with overlapping peptides spanning N protein or S protein for 5 hours in the presence of protein transport inhibitors. ICS and flow cytometric analysis were conducted to measure GZMB-expressing CD8 and CD4 T cells. The five panels in the top showed the gating strategy to identify single, live, CD45+, CD3+CD8+, and CD3+CD4+ T cells. The four panels in the bottom showed representative flow cytometric plots for GZMB+CD8 T cells under different conditions, and correspond to the FACS data in Figure 1i. Negative control (mock stimulation) and positive control (PMA/ionomycin stimulation) were included. ** $p<0.01$; *** $p<0.001$; unpaired student's t test.

Supplementary Figure 3

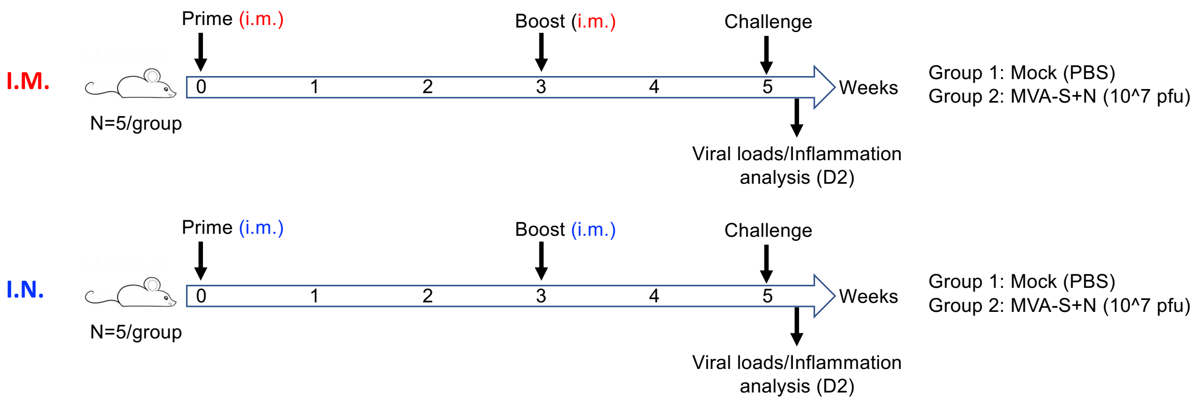

Supplementary Fig. 3. Study design and timeline for vaccination and SARS-CoV2 challenge in mice.

Two groups of WT BALB/c mice (n=5) were prime-boost immunized with mock (PBS) or MVA-S+N (10^7 pfu) at week 0 and 3 via I.M. (top). Another 2 groups of BALB/c mice (n=5) received the same mock or MVA-S+N vaccine (10^7 pfu), respectively, via I.N. route (bottom). Two weeks after boost vaccination (week 5), mice were intranasally challenged with mouse-adapted SARS-CoV2 strain (2X10^4 pfu). Two days after viral challenge, all mice were euthanized and various lung tissues were harvested for analysis of vaccine-induced viral control (viral loads & inflammation) in the lung.
