# Supplementary material for: Mucosal vaccination induces protection against SARS-CoV-2 in the absence of detectable neutralizing antibodies
Source: NPJ Vaccines. 2021 Nov 29;6:139. doi: 10.1038/s41541-021-00405-5 (PMC8630013; doi:10.1038/s41541-021-00405-5)
Supplement: Supplementary file 2 — Reporting Summary [file 41541_2021_405_MOESM2_ESM.pdf]

nature portfolio

Corresponding author(s): Haitao Hu; Tian Wang

Last updated by author(s): 10/09/2021

Reporting Summary

Nature Portfolio wishes to improve the reproducibility of the work that we publish. This form provides structure for consistency and transparency in reporting. For further information on Nature Portfolio policies, see our Editorial Policies and the Editorial Policy Checklist.

Statistics

For all statistical analyses, confirm that the following items are present in the figure legend, table legend, main text, or Methods section.

n/a	Confirmed	
☐	☒	The exact sample size (*n*) for each experimental group/condition, given as a discrete number and unit of measurement
☐	☒	A statement on whether measurements were taken from distinct samples or whether the same sample was measured repeatedly
☐	☒	The statistical test(s) used AND whether they are one- or two-sided<br>*Only common tests should be described solely by name; describe more complex techniques in the Methods section.*
☒	☐	A description of all covariates tested
☒	☐	A description of any assumptions or corrections, such as tests of normality and adjustment for multiple comparisons
☐	☒	A full description of the statistical parameters including central tendency (e.g. means) or other basic estimates (e.g. regression coefficient) AND variation (e.g. standard deviation) or associated estimates of uncertainty (e.g. confidence intervals)
☐	☒	For null hypothesis testing, the test statistic (e.g. *F*, *t*, *r*) with confidence intervals, effect sizes, degrees of freedom and *P* value noted<br>*Give P values as exact values whenever suitable.*
☒	☐	For Bayesian analysis, information on the choice of priors and Markov chain Monte Carlo settings
☒	☐	For hierarchical and complex designs, identification of the appropriate level for tests and full reporting of outcomes
☒	☐	Estimates of effect sizes (e.g. Cohen's *d*, Pearson's *r*), indicating how they were calculated

Our web collection on statistics for biologists contains articles on many of the points above.

Software and code

Policy information about availability of computer code

Data collection	N/A
Data analysis	N/A

For manuscripts utilizing custom algorithms or software that are central to the research but not yet described in published literature, software must be made available to editors and reviewers. We strongly encourage code deposition in a community repository (e.g. GitHub). See the Nature Portfolio guidelines for submitting code & software for further information.

Data

Policy information about availability of data

All manuscripts must include a data availability statement. This statement should provide the following information, where applicable:

- Accession codes, unique identifiers, or web links for publicly available datasets
- A description of any restrictions on data availability
- For clinical datasets or third party data, please ensure that the statement adheres to our policy

All data generated and analysed during this study are included in this published article (and its supplementary information files).

Field-specific reporting

Please select the one below that is the best fit for your research. If you are not sure, read the appropriate sections before making your selection.

☒ Life sciences ☐ Behavioural & social sciences ☐ Ecological, evolutionary & environmental sciences

For a reference copy of the document with all sections, see nature.com/documents/nr-reporting-summary-flat.pdf

Life sciences study design

All studies must disclose on these points even when the disclosure is negative.

Sample size	Sample sizes were not predetermined based on certain statistical methods, and were chosen according to standards of the filed. The number of animals per group in our study (n=5) generated data that gave sufficient statistics for the effect sizes of interest.
Data exclusions	No data were excluded from consideration.
Replication	Multiple levels of replication were included in the study. For biological replicates, five animals were assigned to each group. The data showed cumulative results of all five animals in each group. For technical replicates, in all the assays, at lease duplicates of each sample were included, such as binding ELISA, neutralizing assay, ELISPOT, ICS, PCR etc.
Randomization	Animals were randomly assigned to different groups in this study, including I.M. (mock and vaccine), and I.N. (mock and vaccine).
Blinding	Investigators in this study were not blinded. This is an animal study and we feel blinding is not typically used in the situation.

Reporting for specific materials, systems and methods

We require information from authors about some types of materials, experimental systems and methods used in many studies. Here, indicate whether each material, system or method listed is relevant to your study. If you are not sure if a list item applies to your research, read the appropriate section before selecting a response.

Materials & experimental systems

n/a	Involved in the study	
☐	☒	Antibodies
☐	☒	Eukaryotic cell lines
☒	☐	Palaeontology and archaeology
☐	☒	Animals and other organisms
☒	☐	Human research participants
☒	☐	Clinical data
☒	☐	Dual use research of concern

Methods

n/a	Involved in the study	
☒	☐	ChIP-seq
☐	☒	Flow cytometry
☒	☐	MRI-based neuroimaging

Antibodies

Antibodies used	Anti-SARS-CoV-2 S (GTX632604; GeneTex), anti-SARS-CoV-2 N (MA5-29981; Invitrogen), HRP-linked anti-mouse IgG (7076S, Cell Signaling), HRP anti-mouse IgG secondary antibody (405306; Biolegend), anti-mouse CD45-APC-Cy7 (103116; Biolegend), anti-mouse CD3-PE-Cy7 (100220; Biolegend), anti-mouse CD4-FITC (100406; Biolegend), and anti-mouse CD8-PerCP (100732; Biolegend), anti-mouse GZMB-Pacific Blue (515407; Biolegend)
Validation	The two antibodies detecting SARS-CoV-2 S or N proteins were validated in our western blot assay, where negative control using empty MVA-infected BHK-21 cells were used and showed negative results. HRP-linked anti-mouse IgG (7076S, Cell Signaling) and HRP anti-mouse IgG (405306; Biolegend) were validated at the vendors based on their website. For flow cytometric antibodies, they were validated by immune staining and flow cytometry using control cell populations (subset-depleted mouse cells).

Eukaryotic cell lines

Policy information about cell lines

Cell line source(s)	BHK-21 (ATCC; Cat #: CCL-10); Vero E6 (ATCC; Cat #: CRL-1586)
Authentication	None of these two cell lines were authenticated experimentally by my lab in study. They were obtained from ATCC and look normal based on morphology of cells during culture.
Mycoplasma contamination	Both cell lines were tested negative for mycoplasma contamination.

Commonly misidentified lines (See ICLAC register)	N/A

Animals and other organisms

Policy information about studies involving animals; ARRIVE guidelines recommended for reporting animal research

Laboratory animals	6-week old female BALB/c mice; Jackson Laboratory (Stock No: 000651)
Wild animals	N/A
Field-collected samples	N/A
Ethics oversight	Animal protocol was approved by the Institutional Animal Care and Use Committee (IACUC) at the University of Texas Medical Branch. Animal study was conducted in accordance with the recommendations in the Guide for the Care and Use of Laboratory Animals of the National Institutes of Health.

Note that full information on the approval of the study protocol must also be provided in the manuscript.

Flow Cytometry

Plots

Confirm that:

☒ The axis labels state the marker and fluorochrome used (e.g. CD4-FITC).

☒ The axis scales are clearly visible. Include numbers along axes only for bottom left plot of group (a 'group' is an analysis of identical markers).

☒ All plots are contour plots with outliers or pseudocolor plots.

☒ A numerical value for number of cells or percentage (with statistics) is provided.

Methodology

Sample preparation	Single cell suspensions prepared from lung tissue of mice were used for flow cytometric analysis. Equivalent portions of lung tissues were harvested and digested with collagenase type IV in RPMI Medium. After digestion, lung single-cell suspensions were prepared by passing the lung homogenates through 70µm cell strainers. Red blood cells were removed.
Instrument	BD FACS LSR Fortessa
Software	BD FACSDiva for data acquisition; Flow Jo for data analysis.
Cell population abundance	No sorting was performed in this study.
Gating strategy	Gating strategy involved gating on singlets (FSC-H/FSC-A), live cells (Aqua Blue-), CD45+, CD3+, CD8+ or CD4+ cells. Positive Granzyme B expression in T cells was gated based on cell distribution in FACS plots as well as on negative & positive control cells.

☒ Tick this box to confirm that a figure exemplifying the gating strategy is provided in the Supplementary Information.
